# Correction: Surface core–shell magnetic polymer modified graphene oxide-based material for 2,4,6-trichlorophenol removal

**DOI:** 10.1039/c9ra90041a

**Published:** 2019-06-17

**Authors:** Mei-Lan Chen, Jian-Qing Min, Sheng-Dong Pan, Mi-Cong Jin

**Affiliations:** College of Biology and Environmental Engineering, Zhejiang Shuren University Hangzhou 310015 China; Key Laboratory of Health Risk Appraisal for Trace Toxic Chemicals of Zhejiang Province, Ningbo Municipal Center for Disease Control and Prevention Ningbo China jmcjc@163.com; Ningbo Key Laboratory of Poison Research and Control, Ningbo Municipal Center for Disease Control and Prevention Ningbo China

## Abstract

Correction for ‘Surface core–shell magnetic polymer modified graphene oxide-based material for 2,4,6-trichlorophenol removal’ by Mei-Lan Chen *et al.*, *RSC Adv.*, 2014, **4**, 63494–63501.

The authors apologise that parts of the data presented in Fig. 1, 2 and 5 are incorrect.

The authors have repeated the experiments to provide replacement data for Fig. 1, 2(a) and 5(b, c). The new figures have been reviewed by an expert and are provided below in order to fulfil the journal’s responsibility to correct the scientific record, in accordance with the guidelines provided by the Committee on Publication Ethics (COPE). This correction does not alter the conclusions presented in this *RSC Advances* paper.

(1) The original version of Fig. 1 had been inappropriately modified using photoshop to make the images more appealing. The authors apologise for this and understand that any type of image manipulation is not acceptable. The corrected version of Fig. 1 is shown here:

**Fig. 1:**
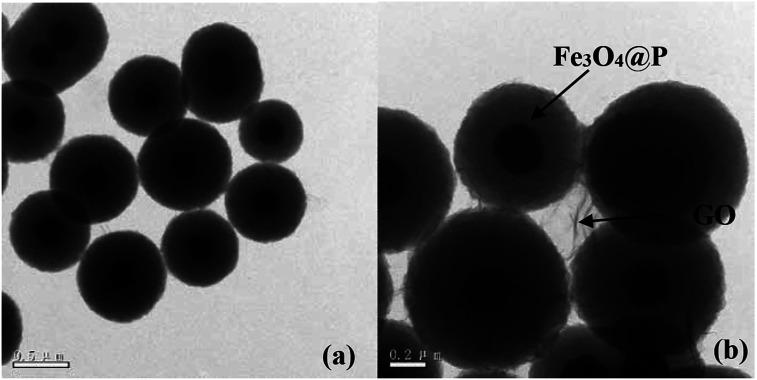
TEM images of (a) Fe_3_O_4_@P, and (b) GO-Fe_3_O_4_@P.

(2) Fig. 2(a) and the related discussion contained some errors in the original manuscript. The original FTIR characterization data was sent to a third party as the authors did not know how to convert the original data into the FTIR graphs. The authors admit that they did not carefully check the results and inadvertently supplied the FTIR data for a different material.

The corrected version of Fig. 2(a) and the corrected sentences are shown here:

Page 63496 (right column), paragraph 2: “As shown in Fig. 2(a), the characteristic peak of Fe_3_O_4_ occurs at 588 cm^−1^. Other typical peaks could be assigned as follows, *ν*(–OH, –NH_2_): 3440 cm^−1^; *ν*(–CH_2_, –CH_3_): 2949 cm^−1^, 2843 cm^−1^; *ν*(–C=O): 1726 cm^−1^; *δ*(–CONH–): 1639 cm^−1^; *δ*(N–H): 1570 cm^−1^.”

Page 63498 (left column), paragraph 2: “Besides, the characteristic peak of N–H bond at 1570 cm^−1^ shifted to 1533 cm^−1^, indicating the formation of hydrogen bond between 2,4,6-TCP and –NH_2_ groups on GO-Fe_3_O_4_@P.^23^ It was worth noting that the peaks at 1452 cm^−1^ and 1392 cm^−1^, due to the skeletal vibration of aromatic C=C bonds, shifted to 1431 cm^−1^ and 1362 cm^−1^, respectively, confirming the π–π stacking interactions were formed between the benzene ring of 2,4,6-TCP and the hexagonal skeleton of the GO sheet of GO-Fe_3_O_4_@P.^24^”

**Fig. 2:**
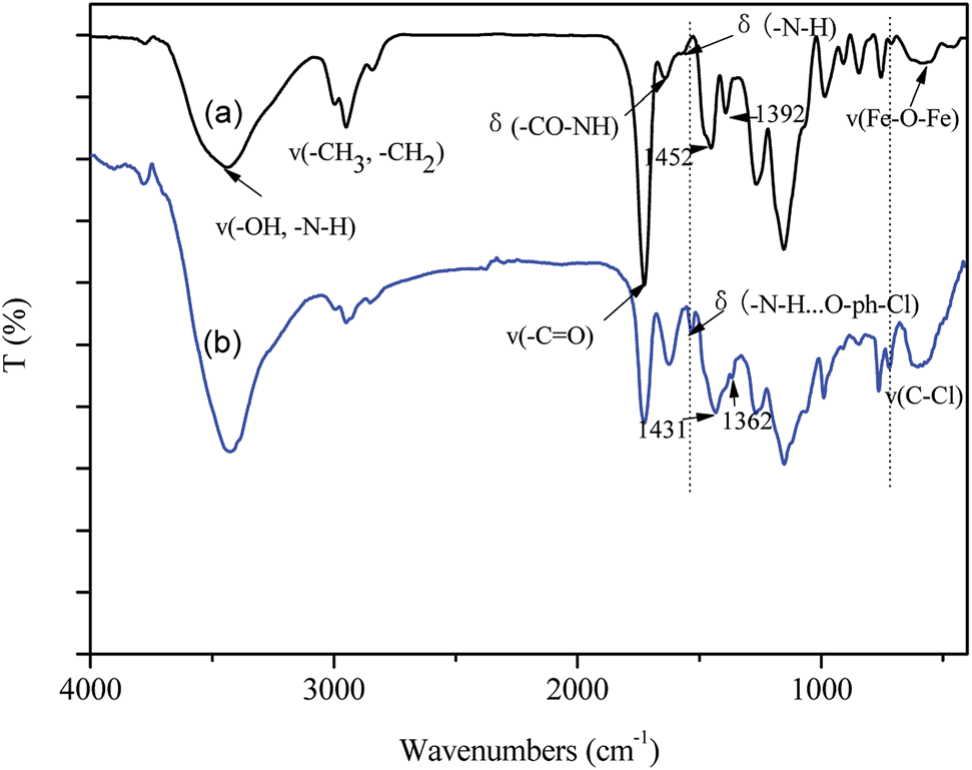
FTIR curves of (a) GO-Fe_3_O_4_@P and (b) GO-Fe_3_O_4_@P-TCP.

(3) Fig. 5(b, c) and the related discussion contained some errors in the original manuscript. The original fluorescence spectroscopy (FL) characterization data was sent to a third party as the authors needed help to convert the original FL data into curves. The authors believe that they have confused the original data and made some mistakes during the process of data transfer. The authors admit that they did not carefully compare the FL curves with the original data.

The corrected version of Fig. 5(b, c) and the corrected sentences are shown here.

Page 63498 (left column) paragraph 2: “The N 1s high-resolution scan of GO-Fe_3_O_4_@P could be deconvoluted into two individual peaks at binding energies of 400.7 eV and 399.3 eV, which could be assigned to N–H, and C–N, respectively. After 2,4,6-TCP adsorption, the binding energy of N–H bond shifted to 400.4 eV, which may be due to the formation of hydrogen bond between 2,4,6-TCP and the amine groups on the surface of GO-Fe_3_O_4_@P.”

Page 63498 (left column) paragraph 2: “The results indicated that the fluorescence intensities at ∼309 nm of GO-Fe_3_O_4_@P were quenched a lot after 2,4,6-TCP adsorption, implying the π–π stacking interaction appeared between 2,4,6-TCP and GO-Fe_3_O_4_@P.”

**Fig. 5:**
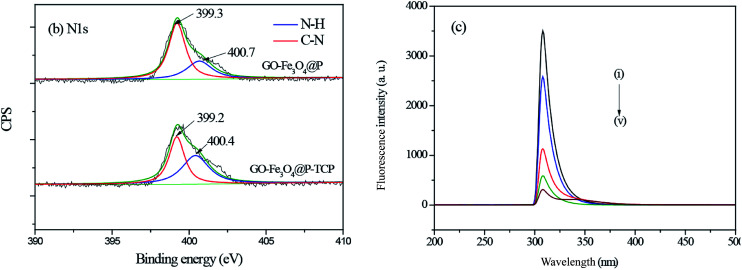
(b) High-resolution scan of N 1s; (c) Fluorescence spectroscopy of: (i) GO-Fe_3_O_4_@P (ii–v) GO-Fe_3_O_4_@P adsorbed with different amount of 2,4,6-TCP (initial 2,4,6-TCP concentrations at 10 mg L^−1^, 100 mg L^−1^, 200 mg L^−1^, and 500 mg L^−1^, respectively).

The Royal Society of Chemistry apologises for these errors and any consequent inconvenience to authors and readers.

## Supplementary Material

